# Contributions of the Guinea Worm Disease Eradication Campaign toward Achievement of the Millennium Development Goals

**DOI:** 10.1371/journal.pntd.0002160

**Published:** 2013-05-30

**Authors:** Kelly Callahan, Birgit Bolton, Donald R. Hopkins, Ernesto Ruiz-Tiben, P. Craig Withers, Kathryn Meagley

**Affiliations:** 1 The Carter Center, Atlanta, Georgia, United States of America; 2 Rollins School of Public Health, Emory University, Atlanta, Georgia, United States of America; (University of Cambridge, United Kingdom)

## Introduction

Infectious diseases have influenced the biological, historical, and political development of the human species more than any other factor: from the outcome of wars to the success of empires, from the pace of technological advance to the structure of society [1]. Dracunculiasis (Guinea worm disease) was considered a mild affliction not warranting a widespread public health campaign. However, examination of the benefits of eradicating Guinea worm disease (GWD) reveals the effort is contributing to development, including contributions to certain Millennium Development Goals (MDGs) [2].

Given the use of the MDGs in the development of global health agendas, it is timely to consider the contributions of neglected tropical disease (NTD) programs, such as the Guinea Worm Eradication Program (GWEP), toward the achievement of the MDGs. The prevention of NTDs, and their cost-effective interventions, fuels long-term economic growth and development, and human advancement [3]. The effort to eradicate GWD is considered one of the most cost-effective health interventions available [2], [4], [5]. The authors provide evidence that concentrated efforts on eradication, elimination, and control of some NTDs can yield far-reaching results, and given these results, stimulate increased efforts toward NTD eradication, elimination, and control among public health advocates, global health entities, and donors.

GWD is a disease of the poor, debilitating many in the most remote and disadvantaged communities in parts of sub-Saharan Africa, where potable water is limited and health care and education are lacking [6]. Endemic GWD transmission is an indicator of extreme poverty [6], [7]. GWD is a preventable, painful, and incapacitating waterborne helminthic disease, which harms health, agriculture, school attendance, and overall quality of life for individuals and communities [7]–[9]. GWD is transmitted when humans drink water, usually from stagnant water sources, containing tiny copepods that have ingested larvae of the parasite. Once consumed, the digestive juices in the human stomach kill the copepods, allowing the larvae to be released and move to the intestinal wall where they migrate to connective tissues of the thorax. Male and female larvae mature and mate 60–90 days after infection. Symptoms appear 10–14 months later when the gravid adult female(s), measuring up to 70–100 cm long, emerges from the skin, causing a painful lesion [10]. When the emerging worm is exposed to water, she ejects hundreds of thousands of larvae into the water to continue the cycle. During emergence, an infected person can be incapacitated for an average of 8.5 weeks [10], [11]. Although rarely fatal, GWD inflicts devastating pain and infection resulting in immobility [8], [12]. The pain is so long-lasting that infected individuals may be incapacitated for up to three months during and after the Guinea worm (GW) emerges [11], [13]–[15]. Other symptoms include nausea, vomiting, diarrhea, and dizziness; secondary bacterial infections can also occur and can lead to arthritis, tetanus, and permanent crippling [8].

There is no cure, vaccine, or immunity after infection [11]. Since there is no evidence that animals are reservoir hosts, the disease is deemed a good candidate for eradication [8]. The global eradication campaign began at the United States Centers for Disease Control and Prevention (CDC) in 1980 and was then adopted as a subgoal of the United Nations International Drinking-Water Supply and Sanitation Decade (1981–1990). In that same year, the decision-making body of the World Health Organization (WHO) adopted a resolution (WHA 34.25) recognizing the decade could be an opportunity to eliminate GWD. Since 1986, The Carter Center (TCC) has led the effort, with the help of the CDC, WHO, United Nations Children's Fund (UNICEF), and many other donors and nongovernmental organizations, to assist national eradication efforts by governments of the countries where GWD is endemic [11]. The GWEP assists ministries of health (MOH) in each endemic country to provide GWD interventions. The GWEP is an eradication effort that relies heavily on behavioral change via health education and interventions. The GWEP has demonstrated that when people are given the proper tools and health education, cases decrease dramatically [16]. The most effective and cost-efficient way to prevent GWD is the promotion of its health campaign coupled with proper and consistent use of filters to remove the copepods from drinking water, case containment, and the application of ABATE, a safe chemical larvicide, to control the copepods [4], [17]–[19].

GWD is an infectious disease categorized as a neglected tropical disease (NTD) [20]. NTDs are among the most common infectious diseases of the world's poorest people [21], [22]. An estimated 1.2 billion people are infected with one or more NTDs [23]. These individuals are among the billion people living on less than $1 per day, a population identified as the “bottom billion” [23]. NTDs are a group of parasitic, bacterial, and viral diseases that cause substantial illness and are a major cause of disease burden that can last decades, impairing children's growth, development, physical fitness, and causing disability and disfigurement [3], [20], [24], [25]. The most common NTDs have relatively high morbidity and low mortality [3], [25]. There are 17 diseases recognized by the CDC as NTDs, of which four (onchocerciasis, schistosomiasis, soil-transmitted helminths, and trachoma) can be controlled, or potentially eliminated, and three eradicated through mass administration of safe and effective medicines complemented with effective interventions, such as behavior change programs (dracunculiasis (Guinea worm), lymphatic filariasis, and taeniasis/cysticercosis) [20], [26]. NTDs are considered “low-hanging fruit” with benefits beyond disease-specific effects, given marked disease burden reduction through cost-effective interventions, mass drug administration, and community-based delivery strategies [4], [27]. The toll of NTDs on individuals, communities, and societies extends beyond physical suffering to significant economic, social, and political consequences, hampering development and trapping the poor in a cycle of poverty and disease [3], [23]. Affected individuals often suffer from multiple NTDs, compounding their individual burden, including disability [3], [26]. This impedes a country's development, in that it impacts children's cognitive development and school attendance and the overall productivity of adults, including agricultural workers, causing disadvantage and hampering a country's ability to address issues of poverty such as unstable or nonexistent public health infrastructure, poor access to clean water sources, disease burden, hunger, and sanitation systems [25].

During the 2000 United Nations Millennium Summit, 189 heads of state committed to improving the health of the global population and promulgated the MDGs [28]–[31]. The MDGs have increased the global commitment to confront the links between poverty and poor health, a link undermining every aspect of life. The MDGs consist of eight goals, encompassing 21 targets addressing hunger and poverty, education, gender equality, child and maternal health, disease burden, the environment, and global partnerships (see Table 1) [32]. NTDs are not specifically addressed in the MDGs, but instead fall under the rubric of “other diseases” in MDG 6: “Combat HIV/AIDS, Malaria and Other Diseases” [32]. An estimated 40 million of the bottom billion are infected with HIV compared to 960 million burdened by NTDs who do not have human immunodeficiency virus (HIV), or who have survived malaria [27]. The combined burden of these parasitic and infectious diseases approaches a loss per year of 56.6 million disability-adjusted life years (DALYs), compared with malaria at 46.5 million and tuberculosis (TB) at 34.7 million DALYs [3], [20], [24], [33].

**Table 1:** The Millennium Development Goals and Targets.

Millennium Development Goals (MDGs)	Targets
Goal 1: Eradicate Extreme Hunger and Poverty	Target A: Halve, between 1990 and 2015, the proportion of people whose income is less than US $1 a day
	Target B: Achieve full and productive employment and decent work for all, including women and young people
	Target C: Halve, between 1990 and 2015, the proportion of people who suffer from hunger
Goal 2: Achieve Universal Primary Education	Target A: Ensure that, by 2015, children everywhere, boys and girls alike, will be able to complete a full course of primary schooling
Goal 3: Promote Gender Equality and Empower Women	Target A: Eliminate gender disparity in primary and secondary education, preferably by 2005, and in all levels of education no later than 2015
Goal 4: Reduce Child Mortality	Target A: Reduce by two thirds, between 1990 and 2015, the under-five mortality rate
Goal 5: Improve Maternal Health	Target A: Reduce by three quarters, between 1990 and 2015, the maternal mortality ratio
	Target B: Achieve, by 2015, universal access to reproductive health
Goal 6: Combat HIV/AIDs, Malaria and Other Diseases	Target A: Have halted, by 2015, and begun to reverse the spread of HIV/AIDs
	Target B: Achieve, by 2010, universal access to treatment for HIV/AIDs for all those who need it
	Target C: Have halted, by 2015, and begun to reverse the incidence of malaria and other major diseases
Goal 7: Ensure Environmental Sustainability	Target A: Integrate the principles of sustainable development into country policies and programs and reverse the loss of environmental resources
	Target B: Reduce biodiversity loss, achieving, by 2010, a significant reduction in the rate of loss
	Target C: Halve, by 2015, the proportion of the population without sustainable access to safe drinking water and basic sanitation
	Target D: By 2020, achieve a significant improvement in the lives of at least 100 million slum dwellers
Goal 8: Develop a Global Partnership for Development	Target A: Develop further open, rule-based, predictable, nondiscriminatory trading and financial systems
	Target B: Address the special needs of least developed countries
	Target C: Address the special needs of landlocked developing countries and small-island developing states
	Target D: Deal comprehensively with the debt problems of developing countries
	Target E: In cooperation with pharmaceutical companies, provide access to affordable essential drugs in developing countries
	Target F: In cooperation with the private sector, make available benefits of new technologies, especially information and communication technologies

Historically, the focus of health policy makers on HIV/AIDS, TB, malaria, and emerging or reemerging diseases has caused funding for NTDs to be overlooked, resulting in deleterious effects on the social and economic well-being of the poorest quintile of populations in the least developed countries [4], [34]. Financing mechanisms emphasize funding for broad sector support rather than disease-specific interventions: in 2010, NTDs received 0.6% of total international development assistance, compared with 37% for HIV/AIDS [3], [35]. Recently, NTDs have received attention from the global health community. For example, in 2008, U.S. President George W. Bush announced the five-year Presidential Initiative for Control of NTDs, committing to make $350 million available for integrated treatment of seven major NTDs. This initiative builds on the United States Agency for International Development's (USAID) existing NTD Control Program, with the potential to treat 300 million people in Africa, Asia, and Latin America [36]. Also, on July 11, 2009 in Accra, Ghana, U.S. President Barack Obama reiterated his administration's pledge of $63 billion for a new Global Health Initiative, which included support for NTDs [37]. In December 2011, the U.S. Congress allocated $89 million for the USAID NTD control efforts in FY2012, which was a $12 million increase over the FY2011 budget for NTDs [38]. Despite the higher burden, new funding, and cost-effectiveness of preventing NTDs, donors and the international health community still direct more resources, funds, and effort to combating the “big three”—HIV/AIDs, tuberculosis, and malaria [3], [21], [26] —than to combating NTDs.

## Contribution of the Guinea Worm Eradication Program toward Achievement of the Millennium Development Goals

Here we describe specific ways the GWEP contributes to achieving certain MDGs:

### MDG 1: Eradicate Extreme Hunger and Poverty

Poverty is not defined by the MDGs; however, an indicator of poverty is a person whose income is less than $1 a day, and extreme hunger is defined as the prevalence of underweight children under five years of age [30]. GWD is not only a symptom of poverty, but also a contributor to poverty [6]. The economic burden of GWD on poor rural communities is severe and compounded by the seasonal nature and transmission patterns of GWD in affected communities [6]. Synchronization of the life cycle of GW with cyclical weather patterns leads to peak transmission periods when unsafe sources of drinking water are consumed [6]. Because of the one-year incubation period, an entire community can be debilitated and unable to work a year later—a period that often coincides with the busiest agricultural seasons [6]. While difficult to determine the exact economic effects of disability among communities of self-employed farmers, its effects on agricultural output can be considerable [14], [39]–[41]. In Ghana, GWD was documented as the major preventable cause of agricultural work loss, since few other diseases coincide with major agricultural activities [14]. In 1989, researchers documented that in one area of Nigeria, a large number of GWD patients were disabled for an average of 12.7 weeks during the yam and rice harvest season [9]. Most of these patients were of school or working age, between 15 and 49 years old [9]. This explains one of the disease's nicknames: “disease of the empty granary” [6], [42]. Before eradication efforts began in the 1980s, some settlements in Nigeria and Ghana experienced GWD in 70% or more of the population during peak agricultural periods, specifically affecting agricultural outputs (MDG 1) and in turn nutrition (MDG 4), as well as overall child health and childcare (MDG 4), school attendance (MDG 2), and other daily social and economic activities [43].

The benefits of more productive labor days, as a result of the reduction in the number of cases of GWD, are not easily measured [13], [14]. However, a 1997 cost-benefit analysis of GWD by the World Bank showed the economic returns compare favorably with those from other health-sector projects [13]. Typically, the World Bank considers an economic rate of return (ERR) in excess of 10% as a benchmark of a sound investment in areas of transport, energy, and agriculture. Based upon GWD eradication by 1998, the GWEP was calculated to have an ERR of 11%, 29%, or 44% if the average period of incapacitation was four, five, or six weeks, respectively, which are conservative calculations considering studies show that individuals are typically debilitated for two to three months [4], [6], [10], [13], [17], [34], [44]. A pilot study in Nigeria provides a subjective perspective in that women of a small farming village attributed their improved standard of living, their ability to work throughout the year (MDG 3), and overall development of their village to the elimination of GWD from their community. They also claimed their families had enough money to pay children's school fees (MDG 2) as well as other expenses [45]. The interruption of GWD transmission allows subsistence farmers to farm and harvest crops, allowing survival from year to year. To the extent the absence of GWD allows farmers to harvest excess crops of commercial value, it can be deemed that eradication of the disease mitigates poverty as defined as income less than a $1 per day.

At the inception of the MDGs in 2000, 14 African countries remained endemic for GWD and reported a total of 75,223 cases [19]. However, actual case numbers were likely higher than reported due to conflict in some countries, specifically Ethiopia and Sudan, which made thorough surveillance difficult [46]. Peace is an essential foundation for improving health, reducing hunger through increased agricultural production, and ending extreme poverty through increased economic opportunities in rural and impoverished areas [46]. “You can bring whatever you like here. If the war continues, it will mean nothing,” said an exasperated village elder from the Nuba Mountains of Sudan [46]. The “Guinea worm cease-fire,” negotiated in 1995 by former U.S. President Jimmy Carter on behalf of the GWEP in Sudan, allowed access to almost 2,000 endemic villages to inaugurate eradication efforts in insecure areas [11], [46]. In 2002, of the endemic Sudanese villages that were accessible, 84% had a resident health worker or volunteer trained in GWD prevention, 85% received health education about the disease, 62% had cloth filters in all households, and 61% had at least one source of clean drinking water [46]. GWD has been and is being used as a diplomatic tool in the region of Sudan to ultimately bring about improved health [4], [11], [15], [46].

### MDG 2: Achieve Universal Primary Education

Those children in sub-Saharan Africa who have access to schooling have experienced poor school attendance associated with GWD [47]. Many students do not receive a consistent education because they are too malnourished to attend class, have an emerging GW and cannot walk to school, or are forced to drop out of school to either assist family members with agricultural or household chores, or replace adults who have GWD and cannot work the fields (MDG 1) [48]. A study in a rural community of southwest Nigeria observed high absentee rates during GWD transmission season. GWD was present in 21% of pupils and was responsible for 25% of days missed throughout the year [40]. However, the provision of alternative potable water sources or access to cloth filters (MDG 7) caused a significant reduction in the prevalence of GWD within three years of the intervention [47]. In ten primary schools located in villages that received borehole wells (MDG 7), pre-intervention surveys conducted during peak GWD season in 1983–1984 showed GWD-related school absenteeism comprised 88% of all absences [47]. The post-intervention survey in 1986–1987 revealed 2.6% of students were absent because of GWD [47]. Another Nigerian study of the impact of 150 borehole wells (MDG 7) provided by the Japan International Cooperation Agency for the GWEP in 135 endemic villages found a decrease of 62.5% in GWD (MDG 6) between 1989–1990 and 1990–1991, compared with an increase of 12% in villages that did not receive boreholes. As a result, school absenteeism decreased by 50% and enrollment increased by 12% in these villages [49]. Subsequently, parents who previously suffered from GWD were healthier post-intervention and were no longer recalling their children from school for domestic support [47]. Similarly, parents were willing to send their children back to school without fear they would become victims of GWD [47]. In communities where there was a high burden of GWD, eradication efforts have increased mobility in parents and children, resulting in a rise in school enrollment and a fall in absenteeism [47].

### MDG 3: Promote Gender Equality and Empower Women

In communities in sub-Saharan Africa, women traditionally perform domestic duties such as childcare, cooking, and cleaning instead of holding jobs outside of the household [48]. Where women perform tasks outside the household, such tasks are typically related to crop production and the subsequent selling of produce [48], [50]. Women in these communities are also responsible for health care and water-related activities, such as water collection, for their households [48]. Using this informal yet established water collection system, the GWEP trained women in community education in order to discourage people with emerging worms from entering water sources, and to conduct GWD surveillance by reporting cases [5]. In the Republic of Benin, networks of women were created for GWD education campaigns to help stop transmission of the disease (MDG 6) using the same community education. Ghana significantly increased its GWD eradication efforts by recruiting and empowering more than 6,800 female Red Cross volunteers to help combat GWD [11], [15]. Also, the GWEP promoted the selection of a female and male volunteer partnership in each endemic village. By 2008, GWEP data showed that 47% of community-based volunteers were women [19]. These women were tasked with various activities related to water—most importantly, the protection of water sources from those infected with GWD. The promotion of women helped improve the quality of water sources for communities that previously lacked access to safe water (MDG 7), and created volunteer opportunities that frequently inspired women to pursue health-related employment (MDG 1) [5].

### MDG5: Improve Maternal Health

Rural African women face many difficulties as a result of living in a disadvantaged environment [50]. Such difficulties include inadequate or nonexistent access to health care, subpar nutritional status due to a harsh agricultural environment that limits crop production, and paucity of potable water [50]. Studies on the effects of GWD on women documented that GWD infection prevented women from performing household management, childcare, and outside employment, resulting in substantial financial losses for the individual and their families [50]. GWD also impacts a woman's overall health and self-care [48]. A qualitative study on women conducted in Oyo and Kwara states in Nigeria showed self-care, defined as washing, eating, and defecating, as well as gender-specific roles like child care and household tasks, were negatively affected when a mother had GWD [48]. Of these functions, self-care was likely to be the most neglected, as the mothers were reluctant to ask for help: they did not wash themselves or their clothes if it meant asking someone to fetch water for them and often ate sparingly because they were not mobile enough to defecate outside [48].

The maternal benefits gained from a community free of GWD are exemplified by a study conducted in three villages in Asa, Nigeria, which formerly had prevalence levels of GWD of 50% or more, but reduced GWD cases to zero (MDG 6) after the provision of safe drinking water sources (MDG 7) [48]. Focus group discussions revealed that after the boreholes were introduced none of the women experienced GWD, and all were aware of the improvement in their own health and well-being. A summary of the mothers' views included quotes such as “When a mother is neat, good looking, wears good clothes, eats good food…she looks healthier. We believe that we are healthier than before because there is no GWD in our community.” Additionally, the women also agreed they were able to better care for their children (MDG 4) and were able to grow and sell produce for their family (MDG 1) [48].

### MDG 6: Combat HIV/AIDs, Malaria and Other Diseases

After the launch of the MDGs, attention focused on HIV/AIDS, tuberculosis, and malaria, but a 2010 study conducted by the World Bank concluded that if the donors shared just 10% of the amount allocated to those “big three” diseases, the most common NTDs could become diseases of the past [24], [26]. Evidence shows people infected with NTDs are more susceptible to coinfection with the “big three” [26]. This evidence also indicates coinfection with one or more NTDs and one of the “big three” may adversely and synergistically affect the patient's prognosis [26], [51]. Therefore, the high prevalence of NTDs in sub-Saharan Africa and its medical significance in terms of affecting the African AIDS epidemic, and endemic malaria and tuberculosis, demands a public health response from the established global community in parallel with efforts to scale up NTD control and elimination [51]. Convincing major stakeholders to establish operational links between HIV/AIDS, malaria, and tuberculosis programs and NTD control, elimination, and eradication activities, especially in sub-Saharan Africa, could increase the efficiency and cost-effectiveness of the “big three” and NTD efforts, and the prognosis of individual patients [51].

The GWEP addresses this public health link via the ministries of health in each endemic country through GWD health education interventions [6]. Since a vaccine does not exist for GWD, the GWEP owes much of its success to behavioral change and health education [6], [52]. By providing education on disease transmission, the GWEP helps people understand how to manage and prevent the disease. When people are given the proper tools, health education, and supervision, there is a dramatic drop in cases (see Table 2) [52]. A review of GWEP data shows marked declines in GWD cases as a result of its efforts. At the inception of the MDGs in 2000, there were 14 GWD-endemic countries reporting a total of 75,223 cases. At the end of 2011, there were only four countries with GWD (South Sudan, 1,030; Mali, 12; Ethiopia, 8; and Chad, 10) reporting 1,060 GWD cases [19]. The GWEP's efforts have averted more than 79 million cases of GWD [53]. With the current resources devoted to the eradication effort, including certification of the global absence of GWD transmission, eradication could be reached before 2015 to coincide with the MDGs [54].

**Table 2:** GWD Eradication Status and MDG Absolute Progress Report through 2010.

Name of Country	Eradication Start Date	Number of Cases Reported at Start Date	Highest Reported Cases	Number of Cases Reported, 2000 (Inception of MDGs)	Number of Cases Reported, 2011	2010 Absolute Progress*** Achieved
Benin	1987	400	37,414	187	0	X
Burkina Faso	1985	458	45,004	1,262	0	X
Cameroon	1985	168	871	3	0	
Central African Republic	1987	1,322	1,322	33	0	
Chad*	1986	314	1,231	0	10	
Cote d'Ivoire	1985	1,889	8,034	290	0	
Ethiopia	1986	3,885	3,885	59	8	X
Ghana	1985	4,501	179,556	6,567	0	X
India	1983	44,818	44,818	0	0	
Kenya	1989	5	53	0	0	X
Mauritania	1985	1,291	8,301	84	0	
Mali	1985	4,072	16,024	292	12	X
Niger	1985	1,373	32,829	1,165	0	
Nigeria	1886	2,821	653,492	7,818	0	
Pakistan	1987	2,400	2,400	0	0	
Senegal	1985	62	1,341	0	0	
Sudan**	1986	822	118,578	51,515	0	
South Sudan**				0	1,030	
Togo	1985	1,456	10,349	828	0	X
Uganda	1985	4,070	126,369	97	0	X
Yemen	1990	0	94	0	0	

* Total includes outbreak in Chad (10) and two cases reported by Ethiopia as imported from South Sudan.

** South Sudan: Became an independent country in 2011. All GWD cases in 2010 and 2011 were reported in South Sudan.

*** Absolute Progress is defined by the MDG Report Card as the biggest positive change on the indicators, regardless of their initial conditions.

### MDG 7: Ensure Environmental Sustainability

GWD is the only disease that can be completely eradicated by the provision of protected water sources, provided these sources are used at all times [5], [18]. The addition of protected water sources in the form of borehole wells or other means can reduce the prevalence of GWD in affected communities from a baseline prevalence of ≥50% to 0% or near 0% in three years or less [47]. For a study conducted in Kwara state of Nigeria, baseline data taken from 1983–1984 indicated 5,134 of the 8,608 (59.6%) individuals surveyed had active GWD [47]. In a post-study evaluation in 1986–1987 after borehole wells were provided, 742 of the 6,425 (11.5%) participants reported GWD: a reduction of 85.5% [47]. Improved water sources also contribute to improved agricultural output (MDG 1) and a reduction of other waterborne diseases (MDG 6), including gastroenteritis, hepatitis, and typhoid fever—all major health problems in Africa [5], [14], [49], [55].

Given the presence of GWD and the absence of safe drinking water are distinct indicators of extreme poverty, a water supply program in which people appreciate the link between the disease and the water supply is an intervention that should be given the highest priority [6], [7], [47]. The GWEP used this concept to further the eradication campaign by advocating with water sector organizations for safer water provision, thereby helping many impoverished communities improve the quality of their water sources [49].

## Discussion

There is little current published research on how combating an NTD such as GWD can result in measureable contributions toward achieving the MDGs [49], [56]. The MDGs were designed to help all levels of poverty, in particular the poorest of the poor. The GWEP addresses the most disenfranchised extreme bottom of this poverty. These populations are so impoverished they often do not have access to rudimentary infrastructures, such as health clinics, schools, and simple technologies that the MDGs use as indicators of progress. This makes quantifying beneficial impacts difficult for some of the MDGs. While data are currently unavailable or insufficient to establish a link between the GWEP and MDG 4 and 8, the GWEP has shown that even in poor, small, and remote villages, health interventions based in the community and implemented by village volunteers are feasible and successful [8], [49].

These overall achievements are having an impact on the health and development of this population and are thus contributing toward the attainment of some MDGs. Eight of the 11 countries in the 2010 MDG progress report that succeeded in obtaining “absolute progress” received assistance from the GWEP and interrupted transmission of GWD (see Table 2). Our research indicates endemic communities would have continued to suffer from increased disease burdens, reduced economic productivity, poor school attendance, and scarce potable water sources had the GWEP not intervened. In the absence of the GWEP, an estimated 3.5 million new GWD cases would have occurred annually for a cumulative total of 79.2 million cases from 1986–2010 [53], [57]. GWD is expected to be the first parasitic disease to be eradicated, and the first disease to be eradicated without a vaccine or cure [4], [11]. Eradication is the ultimate “sustainable” improvement in public health. The case reduction and eventual worldwide eradication of GWD contributes discernible progress to global health for eternity [58].

Eradicating GWD has become a powerful, broad-based “engine for development” by improving agricultural production and school attendance, developing village-based surveillance for GWD as well as other diseases such as malaria, building local capacity by training village volunteers, providing on-the-job managerial experience, promoting clean drinking water, and providing a tangible “peace dividend” in some areas of conflict [5], [11], [15]. At a total cost to date of about $350 million for the global eradication campaign, excluding the cost of safe water supply, the financial cost of the campaign per case averted is estimated to be $3.47 [5], [55]. Additionally, the established GWD community-based surveillance and health education delivery systems are now poised to deliver other health interventions. For example, the Geographic Information System (GIS) database, which was established and developed by UNICEF for the Burkina Faso GWEP, is now being used for other UNICEF-supported health, nutrition, education, water, and sanitation interventions [59]. And village-based GWD surveillance, which was nonexistent in countries such as Ghana and Nigeria at the start of the GWEP, is now being used for reporting other diseases such as tetanus, lymphatic filariasis, and leprosy [6], [44]. The socioeconomic benefits of this global health effort will accrue forever, once GWD eradication is achieved [8]. Conclusively, we believe greater efforts to eradicate, eliminate, and control NTDs could make significant contributions toward achieving the MDGs.

**Key Learning Points**

- Given the prominence of the MDGs in the development of global health agendas, it is important to consider greater implementation of NTD strategies, such as the GWEP, in achieving the MDGs. This publication should stimulate interest among public health advocates, global health entities, and donors, as well as further intellectual discussion, additional research efforts, and publications on alleviating and preventing NTDs as a strategy in achieving MDGs.
- The GWEP has decreased GWD morbidity. Of the countries being measured by the MDGs, eight of the 11 countries showing Guinea worm case reductions also showed clear progress toward achieving the MDGs in receiving the grade of “absolute progress” in the 2010 MDG report card.
- Emerging evidence shows people infected with NTDs are more susceptible to becoming infected with the “big three.” Evidence also indicates that coinfection with one or more NTDs may adversely and exponentially affect the patient's prognosis of having one of the “big three” diseases.

**Five Key Papers in the Field**

- Hopkins DR, Ruiz-Tiben E, Downs P, Withers PC, Maguire JH (2005) Dracunculiasis eradication: the final inch. Am J Trop Med Hyg 73: 669–675.
- Hotez PJ, Molyneux DH, Fenwick A, Ottesen E, Ehrlich Sachs S, et al. (2006) Incorporating a rapid-impact package for neglected tropical diseases with programs for HIV/AIDS, tuberculosis, and malaria. PLoS Med 3: e102. doi:10.1371/journal.pmed.0030102
- Barry M (2007) The tail end of guinea worm — global eradication without a drug or a vaccine. N Engl J Med 356: 2561–2564.
- Watts S (1998) Perceptions and priorities in disease eradication: dracunculiasis eradication in Africa. Soc Sci Med 46: 799–810.
- Peries H, Cairncross S (1997) Global eradication of Guinea worm. Parasitol Today 13: 431–437.
